# Justice at Last to Dr. Beale

**Published:** 1856-01

**Authors:** 


*Justice at last to Dr. Beale.*—The law's delay "has never been more unhappily illustrated than in the case of the unfortunate Dr. Beale. Party politics have interfered with the demands of justice. One governor could not pardon him on the eve of an election, lest it might influence the ballot box on that important occasion, and another could not liberate him until a second election had so completely destroyed the prospects of his party as to absolve him from the necessity of courting votes.

The whole history of this man's trial and incarceration, leaves an indelible blot upon the jurisprudence of Pennsylvania. He fell a sacrifice to the misguided justice of the chief city of the state. An incompetent jury pronounced him guilty on the testimony of a girl, by her own confession, delirious from ether. There was not the shadow of real evidence adduced by the prosecution, and the unhappy victim stands acquitted in the minds of all men of common sense and average justice of the atrocious charge brought against him. Never before was there so shameful a neglect of the rules of evidence, except, perhaps, in the High Court of Commission in the Assizes over which the notorious Jeffrey presided. That a man should be convicted of so grave a charge on the simple assertion of a woman, whose statement confesses to an intoxication, so thorough as not to be sensible to the most serious affront which could be offered her, and whose story abounded in absurd contradictions, sufficient entirely to have ruled out her testimony, on a question involving the smallest amount of property cognizable by the laws, is an astounding evidence of the facility with which juries are ruled by public opinion. This, perhaps, was to be expected, but that the judge should have permitted such testimony to be taken, that he should have allowed the jury to come to a decision without knowing whether the woman was still a virgin or not, whether any of the signs of violation were present, is a dereliction of duty upon which we will not trust ourselves to comment on. Then that two governors in succession could be found, so indifferent to the demands of justice, so careless of the sufferings of an innocent man and his troubled family, as to allow him to languish so long in confinement, is a painful example of magisterial inefficiency which we hope never to see followed. It cannot be pretended that any new light has been thrown upon the question by this cruel delay. The governor, himself, in the most disreputable document which extends the pardon, acknowledges that he believes the prisoner innocent of the charge, on the ground of insufficient testimony adduced on the trial, a fact which he might have ascertained on the first day in which he assumed the chair of state, as fully as this late hour, when unmerited disgrace and undeserved suffering has bowed the hearts of the defendant and his family.

We have used the phrase "disreputable document," in speaking of the governor's pardon, with a full sense of the meaning of the words, and we need only call our reader's attention to the singular discrepancy in the two paragraphs of this remarkable paper quoted below, to justify our strong language. Though acknowledging the man's *innocence,* he has the impudence to read him a lecture as though he were guilty. Not willing to lose the political influence of the enemies of the persecuted man, he cants about "the moral reform of the prisoner," and panders in the most abject manner, to this "public sentiment," to which he considers "ample satisfaction has been rendered;" *ample satisfaction* for what? An innocent man has rendered ample satisfaction for a crime which their majesties, a fraction of the people, have seen fit to imagine. Our cheeks burn with shame, to be compelled thus openly to acknowledge the charge, so often urged against by the European enemies of republicanism, that in this country public opinion often becomes a despot, compared to which the Czar himself is a mild and powerless ruler. That a man in so high position, however, should thus shamelessly avow himself, so submissive a slave to this irresponsible power, fills us with horror and disgust.

It is true, he quotes these objectionable phrases as the opinion of the Board of Inspectors of the Philadelphia County Prison, but they are used as a part of the reason for the man's acquittal, linked together among the influential "whereases," which have induced the act of executive *clemency*.

We wish once more, to disclaim all personal feeling in this matter. When we were called upon a year ago to comment on the finding of the jury, we explicitly declared that we had no personal acquaintance with this unfortunate victim of public opinion. We renew that assertion now. We have never seen or spoken with Dr. Beale; but believing him, as we have ever believed him, altogether innocent of the crime laid to his charge, we have had the honesty to express our opinion in such language, as the atrocity of the persecution seemed to demand.

He had received communications from about one hundred and forty dentists and twenty-three physicians of this city and the country, stating their belief that testimony as to matters transpiring under the influence of ether is unsafe and unreliable; from a number of other physicians named, that they believed him innocent; from a number of the bar, and citizens of various states, including the names of governors, attorneys-general, etc., that they believed he was convicted on insufficient testimony; from a number of clergymen, that they believed him innocent; from the Mayor of Philadelphia, and fifty members of the Philadelphia city council; from members of the legislature, judges of the supreme court; editors of Philadelphia newspapers, and five thousand others citizens of Pennsylvania and New York, with five of the jury on the trial, all asking his pardon. After enumerating all these facts, the governor says:

"And whereas, the board of inspectors of the said Philadelphia county prison, (as appears by their communication on file in the office of the secretary of the commonwealth,) have unanimously recommended the pardon of the said Dr. Stephen T. Beale, because, in their opinion, the end contemplated by the law in the moral reform of the prisoner has been attained—because full and ample satisfaction has been rendered to public sentiment by the imprisonment he has already undergone—because his health is undoubtedly breaking down under the sufferings of body and mind which he has already endured, and because the destitute condition of his aged parents and bereaved and sorrowing wife and children imperatively demand the presence and support of their son, husband and father.

"And whereas, after a full and careful examination of the facts and evidence in the case, aided by the scientific discussion to which it has given rise, (without any intention to reflect upon the prosecutrix, who no doubt testified to what she believed did occur—nor to impugn the integrity of the learned judge who tried the case, nor the honesty of the jury who convicted the prisoner,) *I am now satisfied* that the defendant, Dr. Stephen T. Beale, is *not guilty* of the crime whereof he stands charged, and was convicted upon evidence unreliable in its character, and insufficient in amount.

"I do, therefore, in consideration of the premises, pardon the said Dr. Stephen T. Beale of the crime whereof he is convicted as aforesaid, and he is hereby fully pardoned accordingly."

